# Supplementary material for: The Chemistry of Spinel Ferrite Nanoparticle Nucleation, Crystallization, and Growth
Source: ACS Nano. 2024 Mar 25;18(14):9852–70. doi: 10.1021/acsnano.3c08772 (PMC11008356; doi:10.1021/acsnano.3c08772)
Supplement: Supplementary file 1 — nn3c08772_si_001.pdf [file nn3c08772_si_001.pdf]

Supporting Information:

The Chemistry of Spinel Ferrite Nanoparticle Nucleation, Crystallization and Growth

Henrik L. Andersen,[a,b]* Cecilia Granados-Miralles,[c] Kirsten M. Ø. Jensen,[d] Matilde Saura-Múzquiz,[b] and Mogens Christensen[e]

[a]Instituto de Ciencia de Materiales de Madrid (ICMM), CSIC, Madrid, 28049, Spain
[b]Facultad de Ciencias Físicas, Universidad Complutense de Madrid, Madrid, 28040, Spain
[c]Instituto de Cerámica y Vidrio (ICV), CSIC, Madrid, 28049, Spain
[d]Dept. of Chemistry and Nanoscience Center, University of Copenhagen, København Ø, 2100, Denmark
[e]Dept. of Chemistry and Interdisciplinary Nanoscience Center, Aarhus University, Aarhus C, 8000, Denmark

***E-mail**: henrik.andersen@csic.es

In situ synchrotron PXRD data

In situ PXRD data - $MnFe_2O_4$

$MnFe_2O_4$ - 200 °C

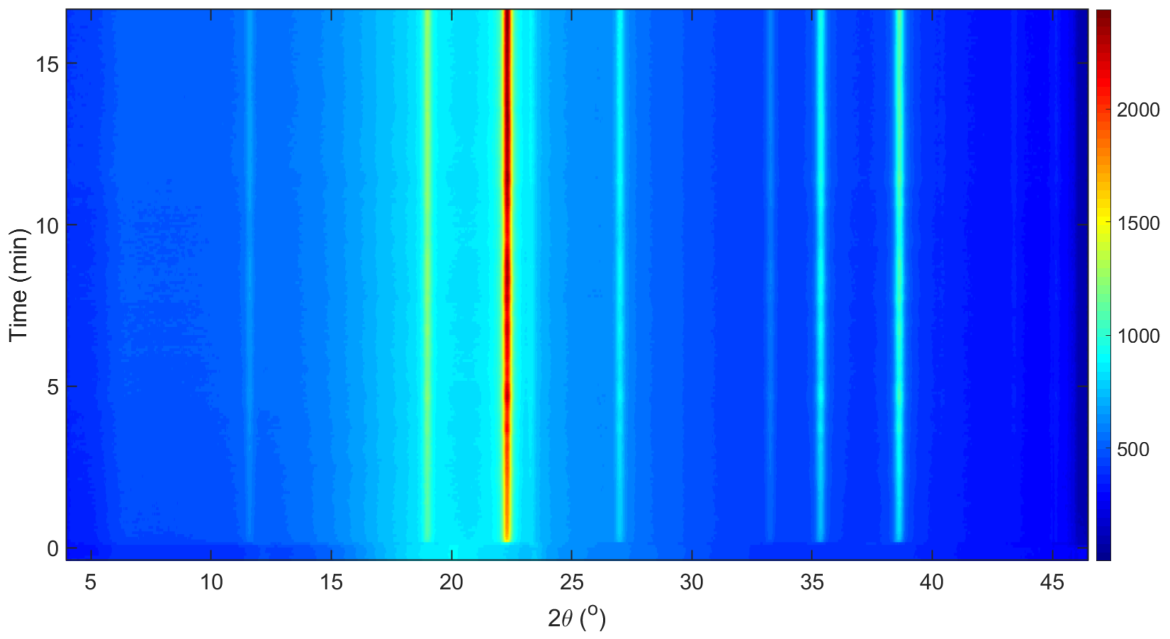

$MnFe_2O_4$ - 250 °C

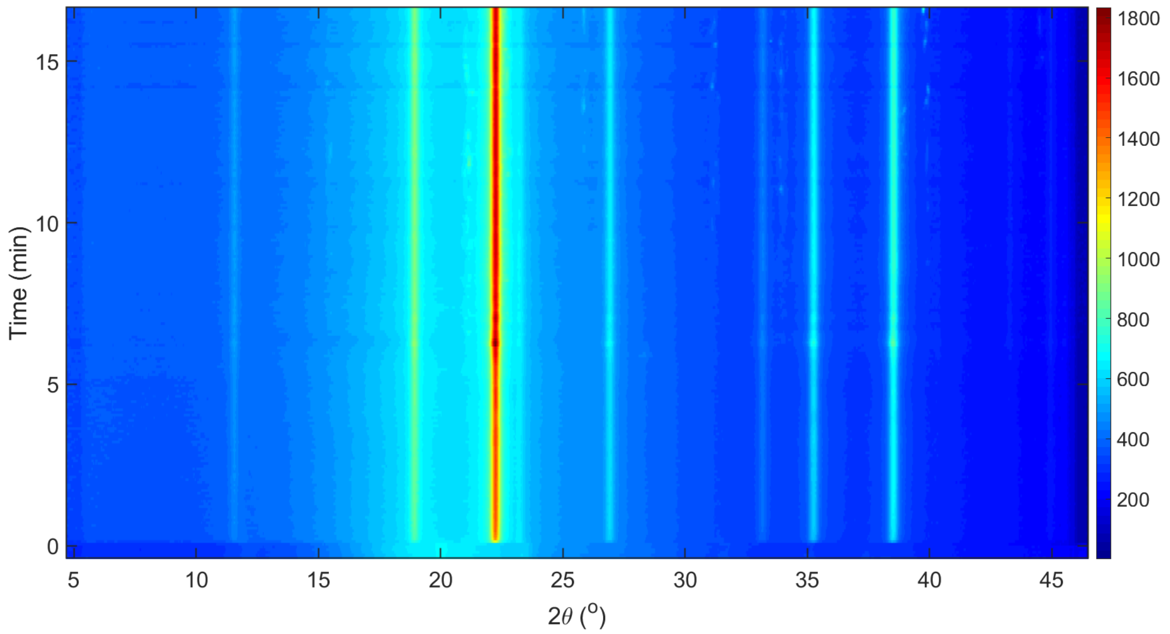

$MnFe_2O_4$ - 300 °C

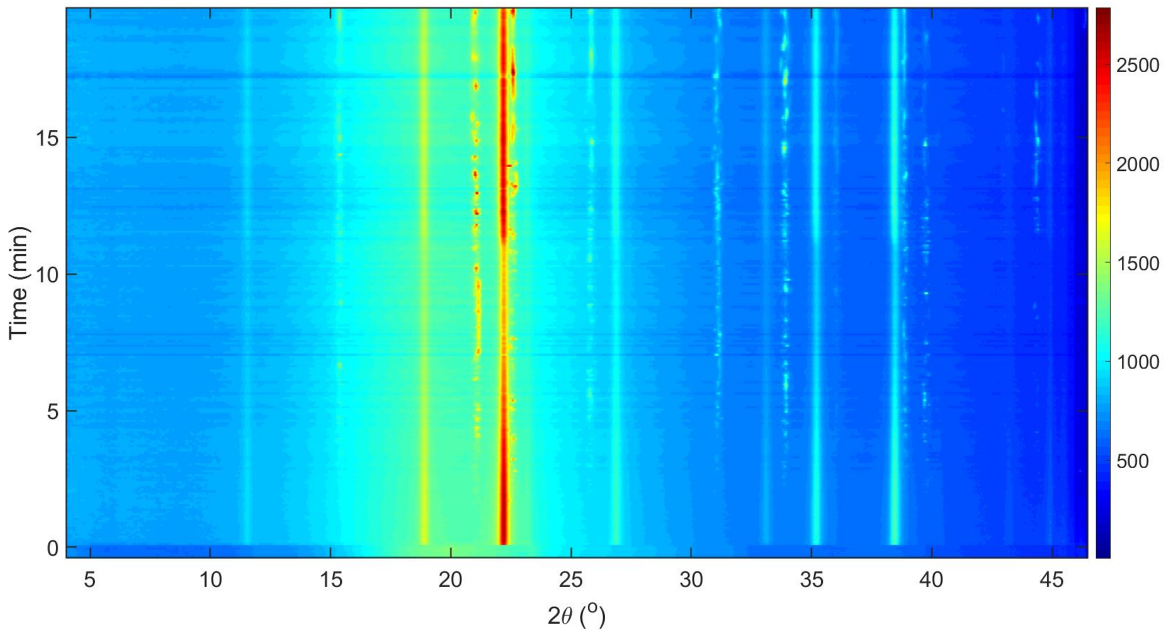

In situ PXRD data - $CoFe_2O_4$

$CoFe_2O_4$ - 170 °C

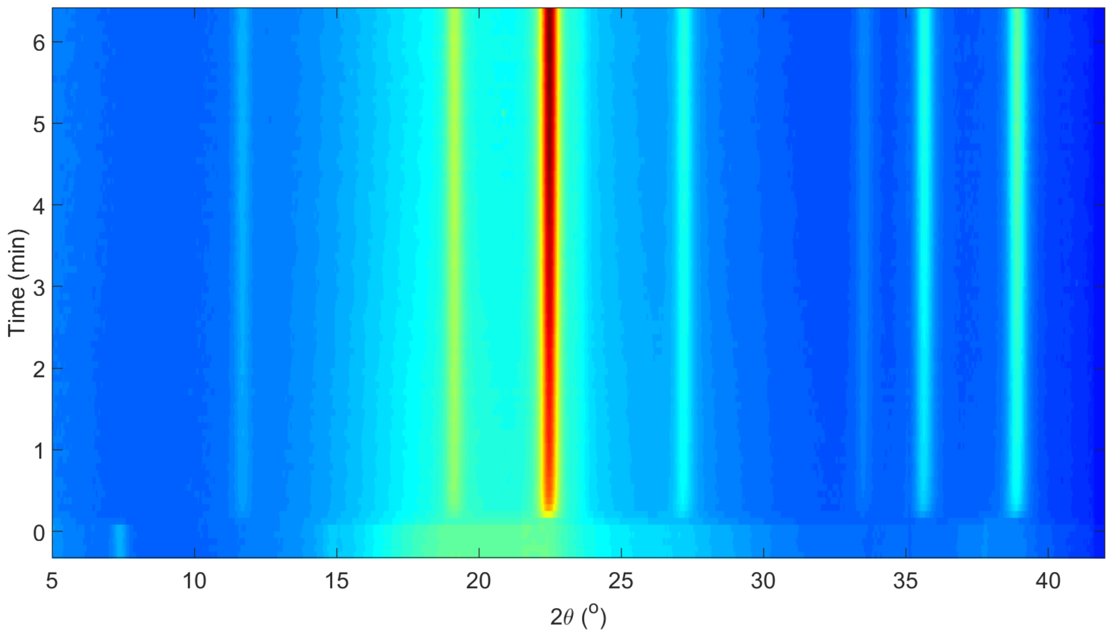

$CoFe_2O_4$ - 230 °C

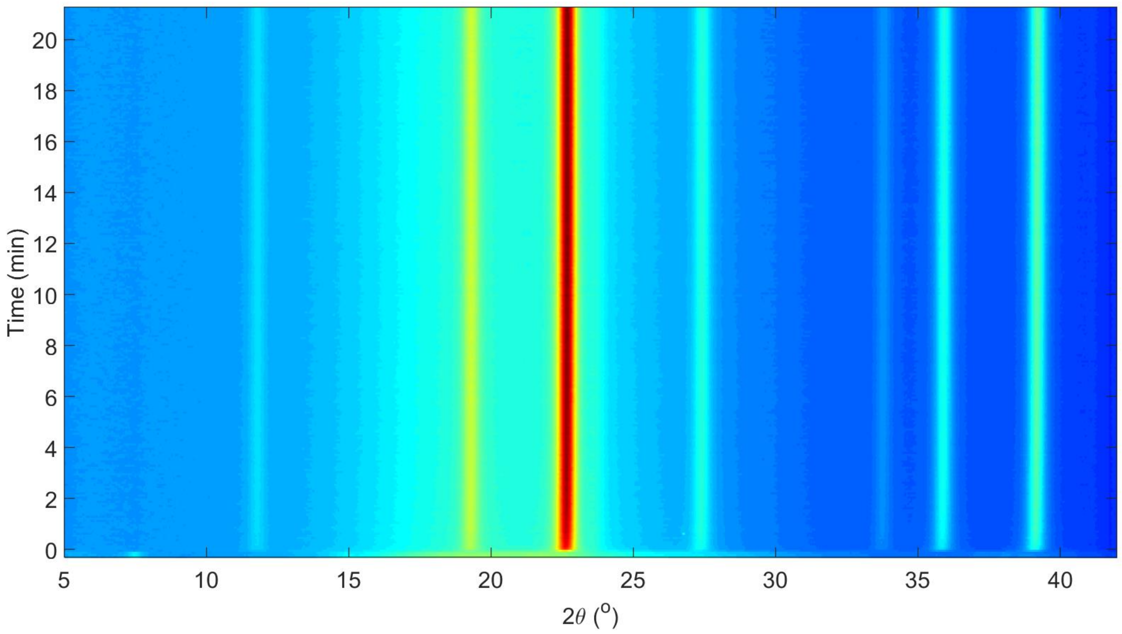

$CoFe_2O_4$ - 270 °C

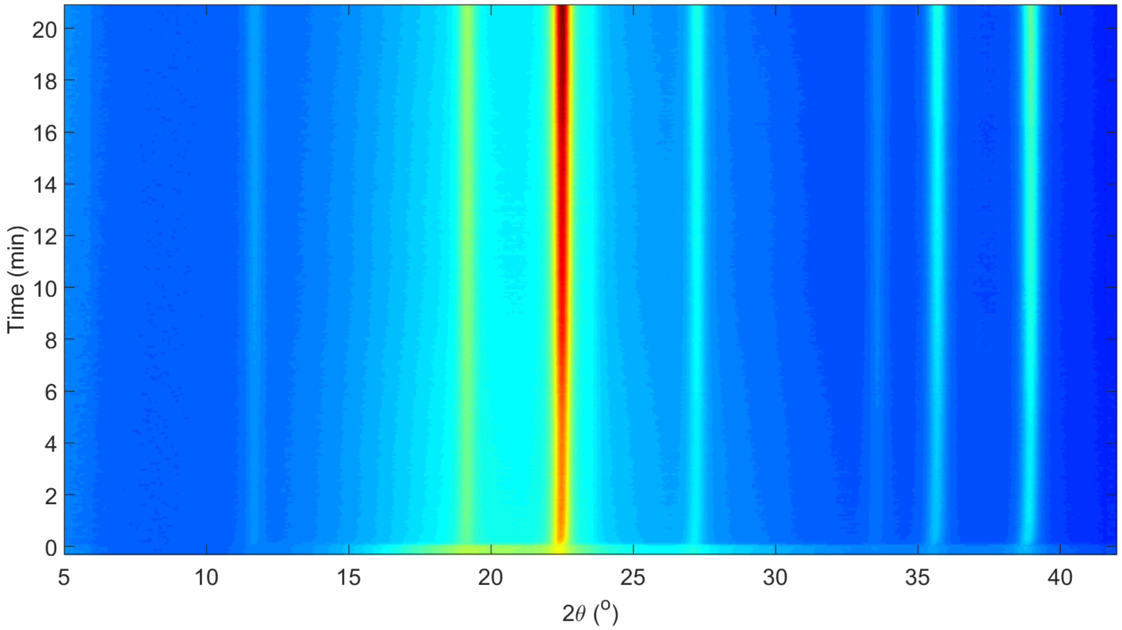

$CoFe_2O_4$ - 320 °C

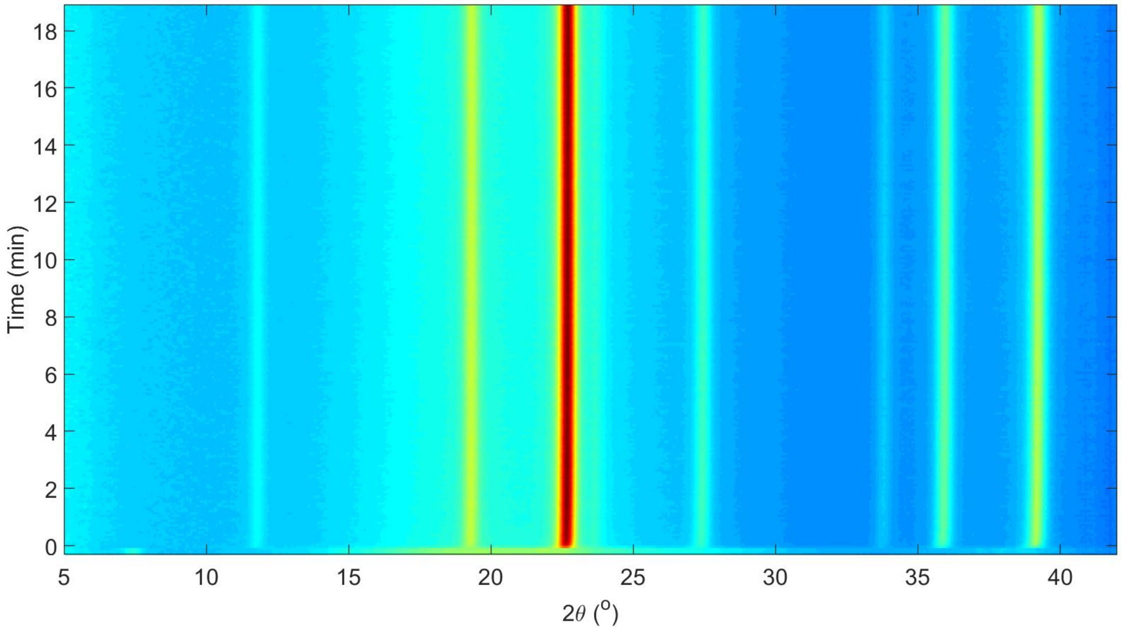

$CoFe_2O_4$ - 370 °C

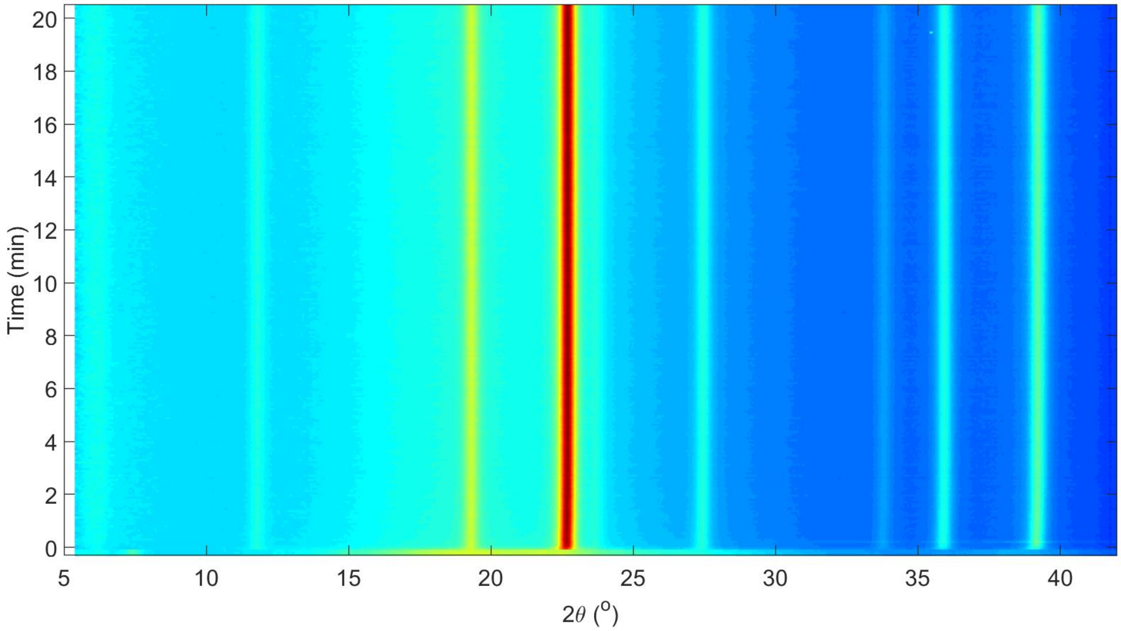

$CoFe_2O_4$ - 400 °C

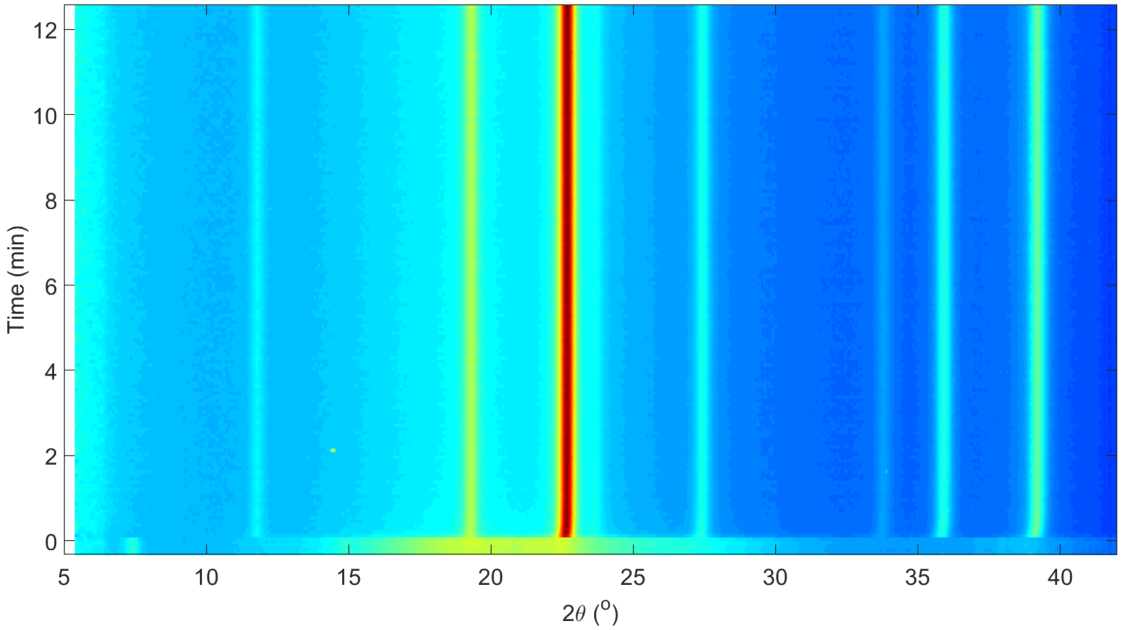

In situ **PXRD data -** $NiFe_2O_4$

$NiFe_2O_4$ - 150 °C

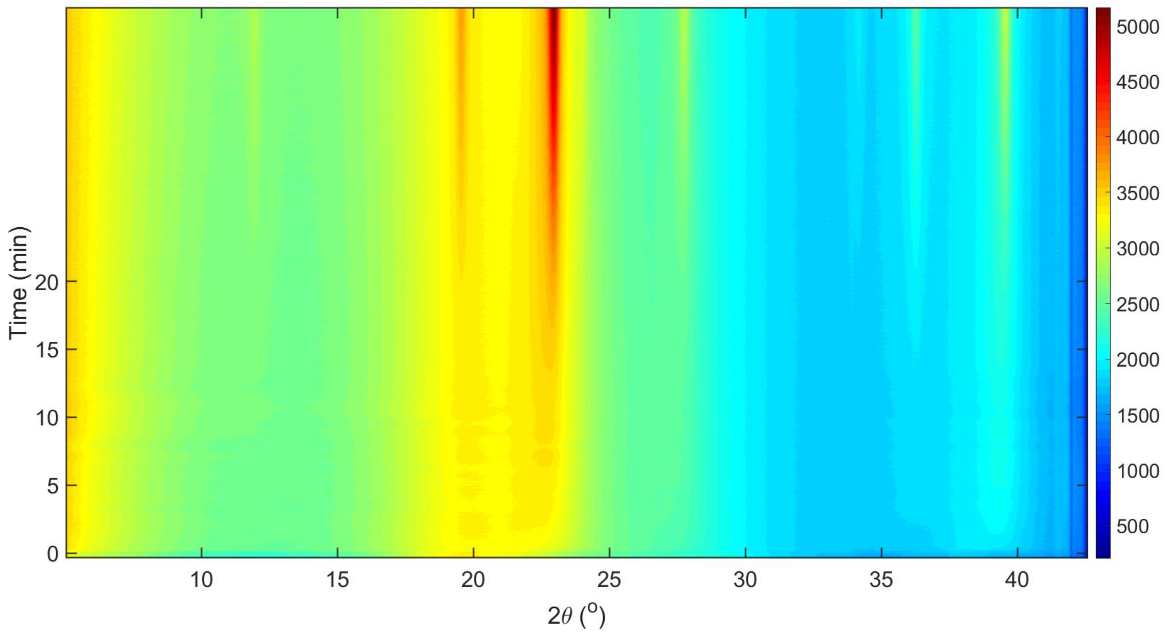

$NiFe_2O_4$ - 200 °C

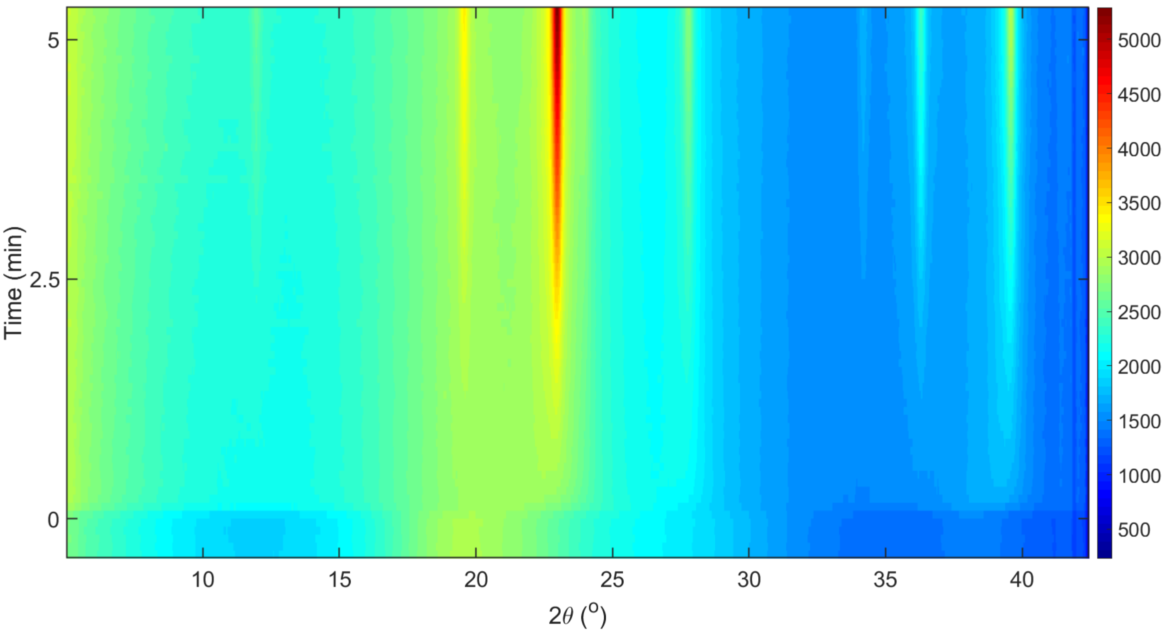

$NiFe_2O_4$ - 250 °C

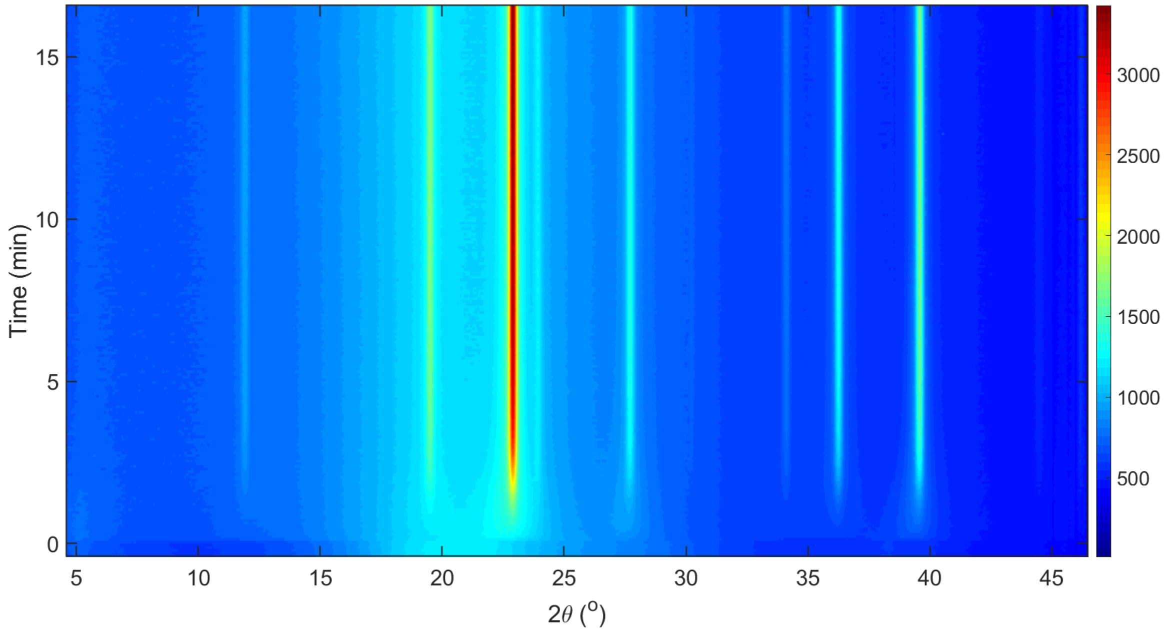

$NiFe_2O_4$ - 300 °C

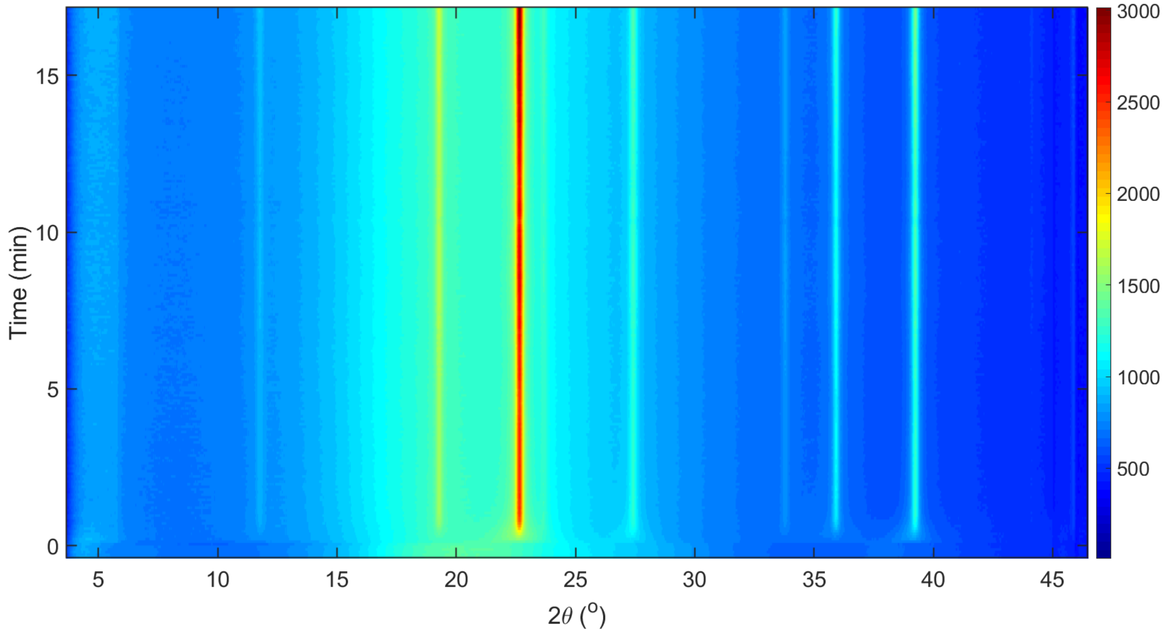

$NiFe_2O_4$ - 350 °C

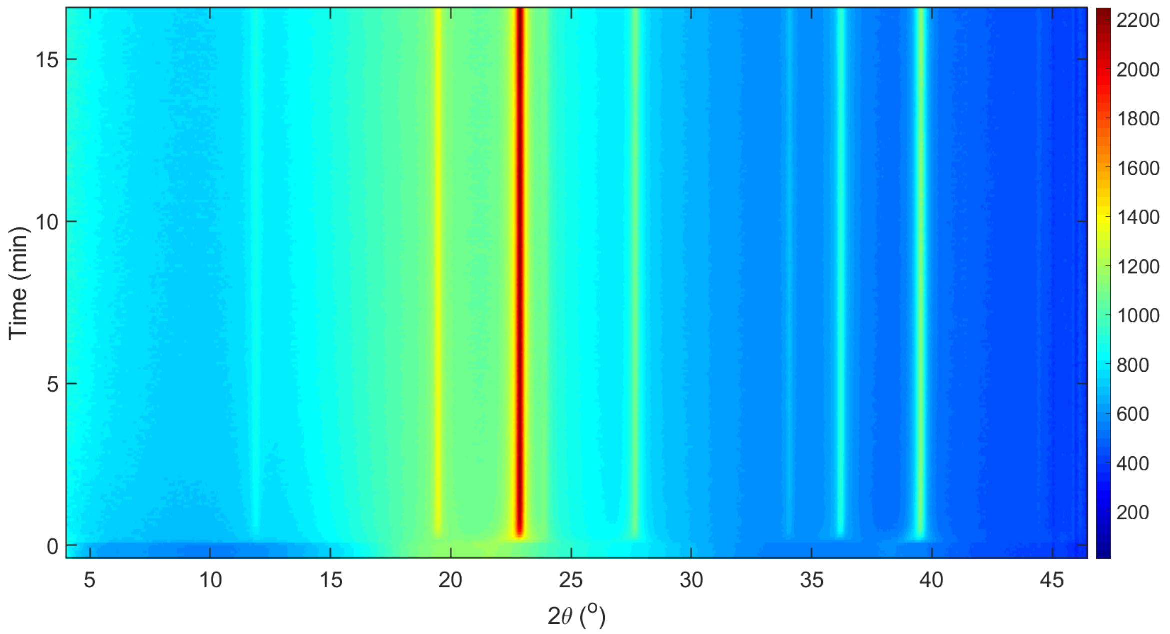

$NiFe_2O_4$ - 400 °C

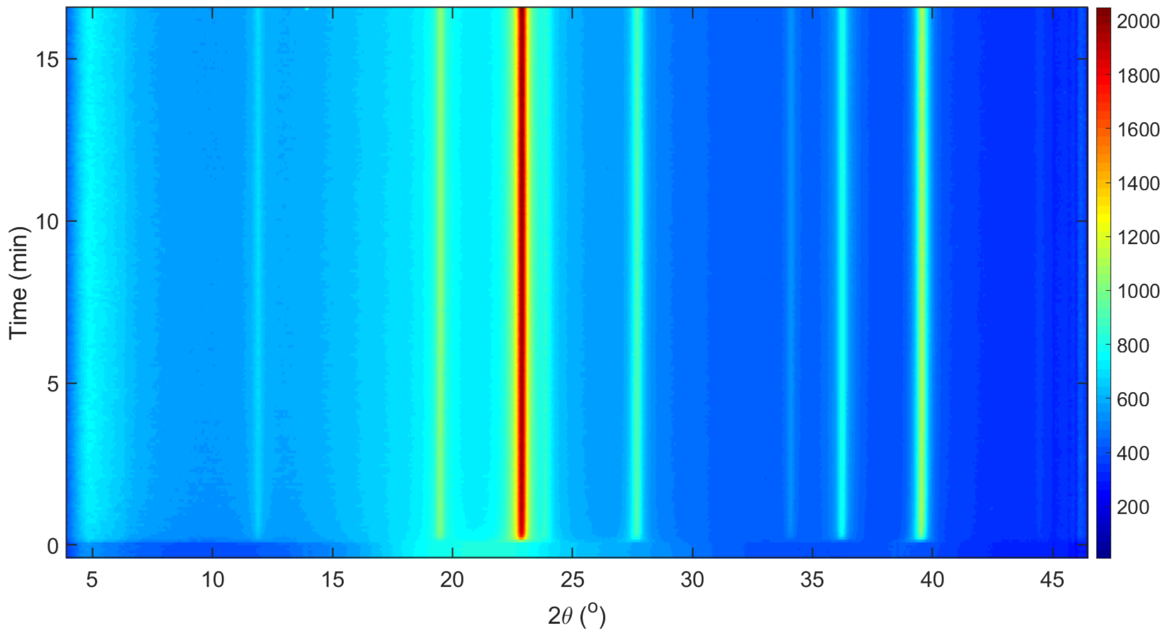

In situ PXRD data - $ZnFe_2O_4$

$ZnFe_2O_4$ - 200 °C

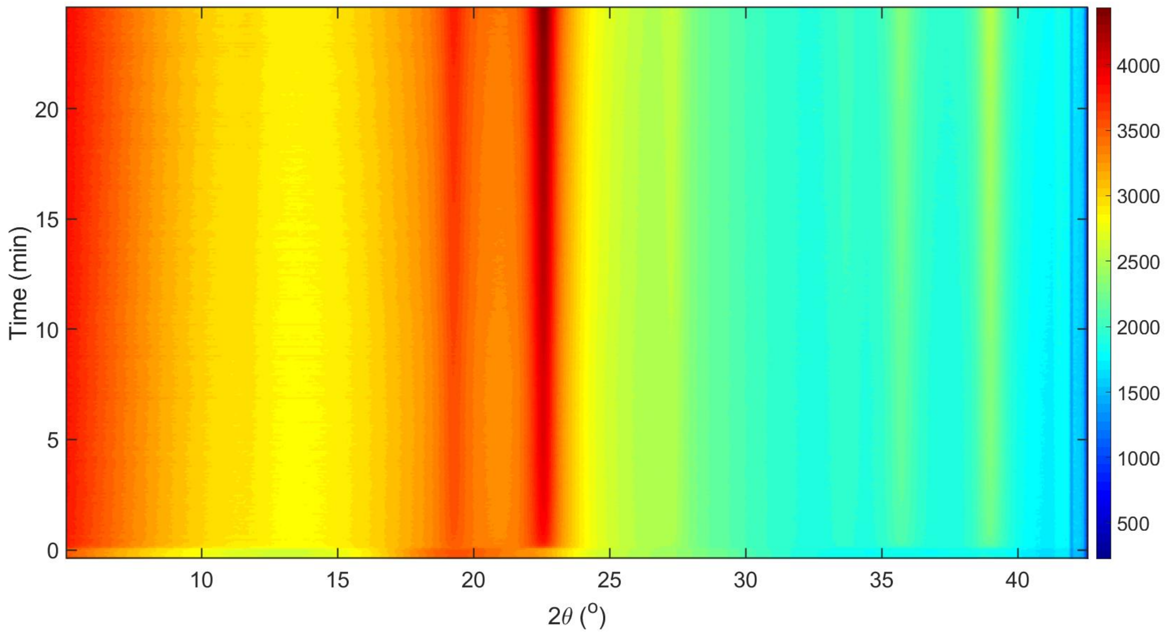

$ZnFe_2O_4$ - 250 °C

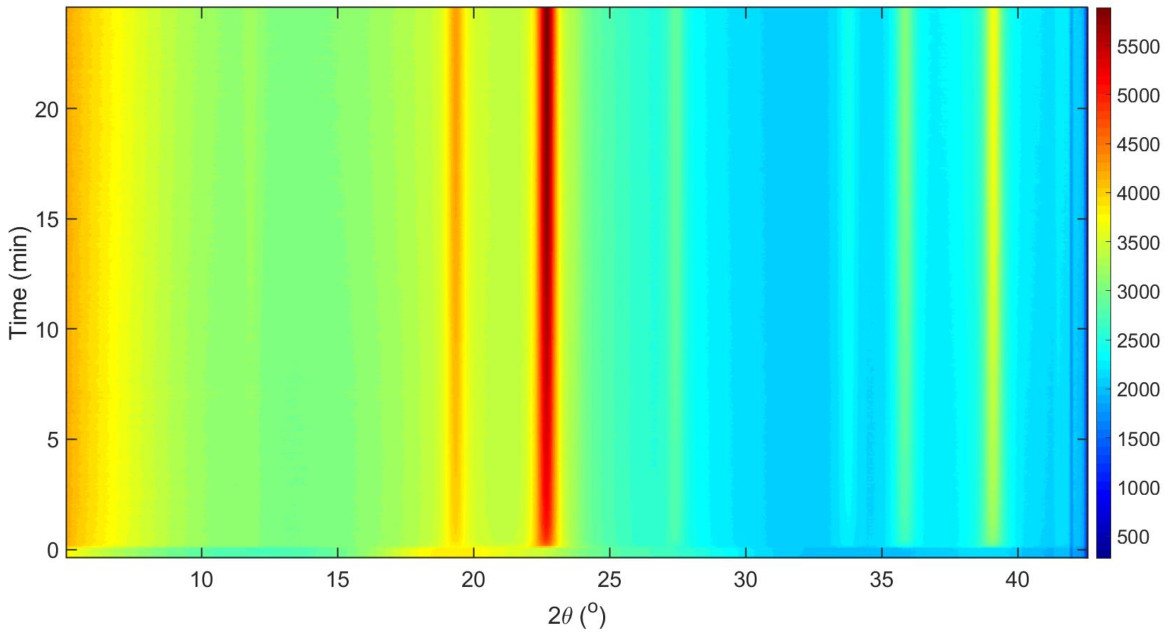

$ZnFe_2O_4$ - 300 °C

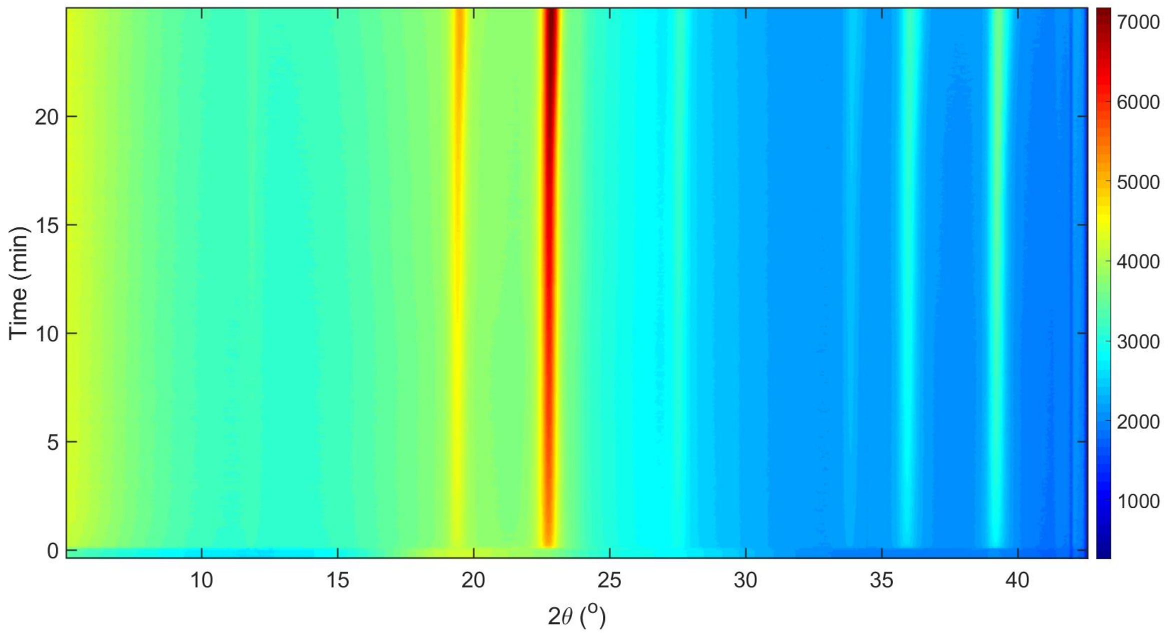

$ZnFe_2O_4$ - 350 °C

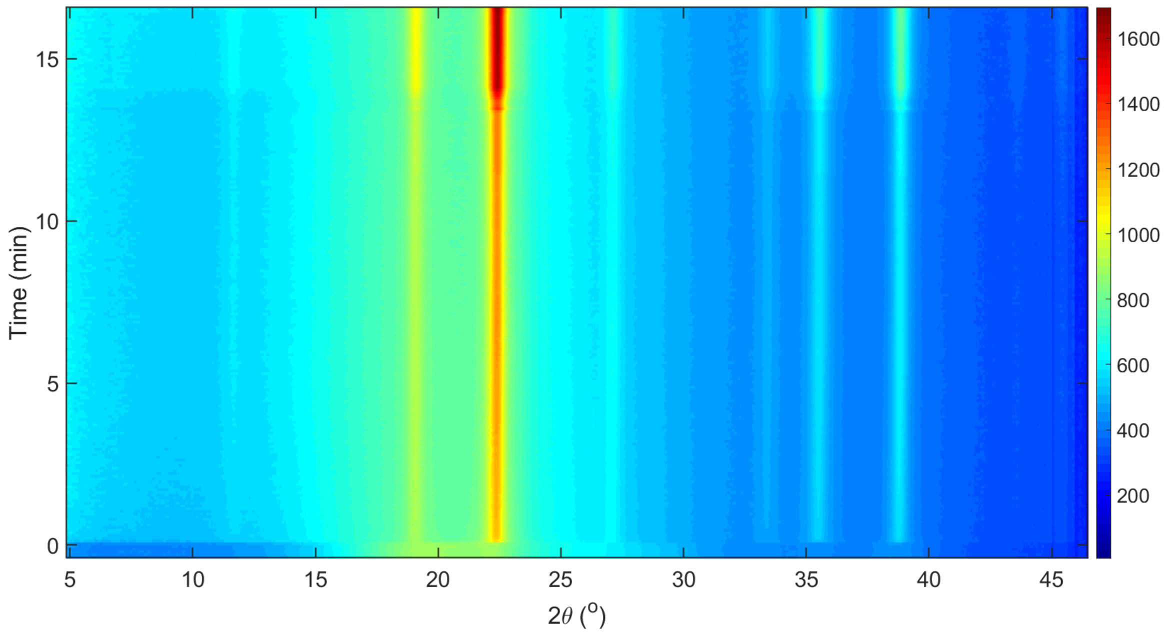

$ZnFe_2O_4$ - 400 °C

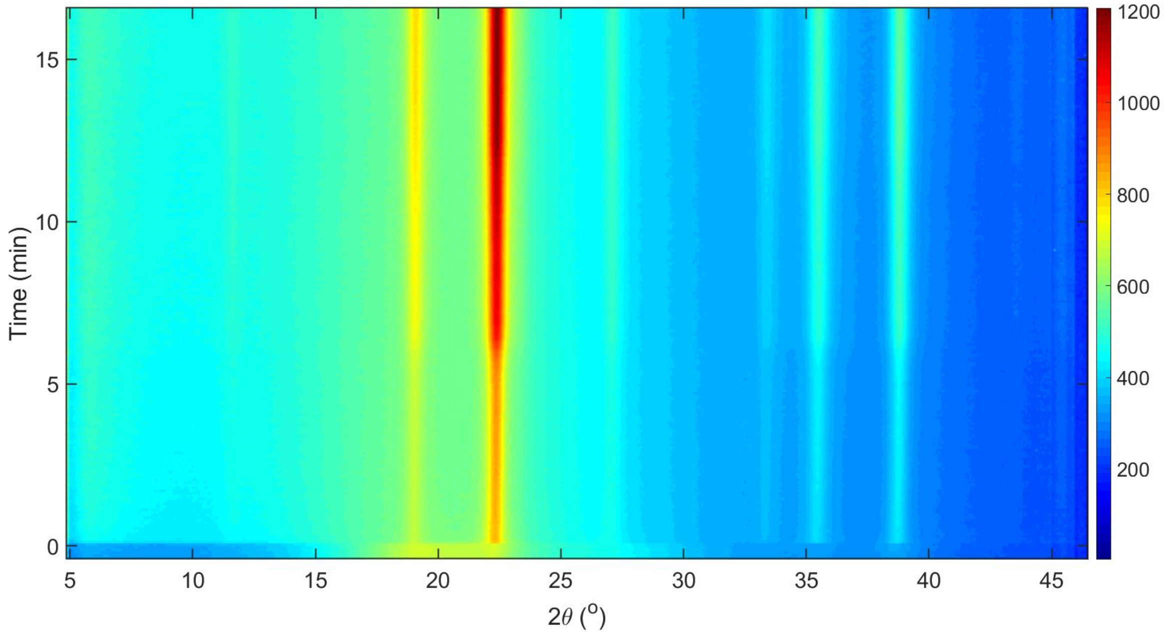

In situ PXRD - Unit cell parameters

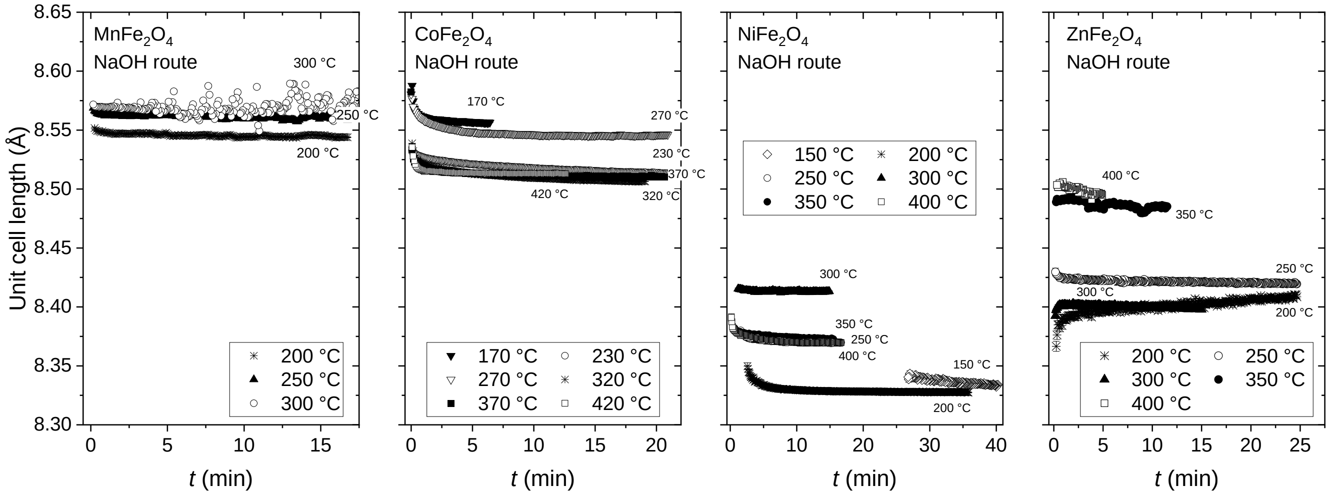

The effect of size distributions and volume averaged mean crystallite dimensions

The Scherrer analysis of the peak shapes (TCH pseudo-Voigt function) implemented in the refinement procedure, yields the volume-averaged mean dimension of the coherently scattering crystalline domains due to the inherent volume weighting of the PXRD technique (*i.e.* smaller crystallites are underweighted compared to larger ones as their scattering contribution increases with <*D*>^{3}). Considering this along with the fact that real nanoparticle systems are never fully monodisperse and rarely exhibit perfect Gaussian size distributions, the crystallite size (be it number- or volume-averaged) typically differs from the median size. This is illustrated in Figure 1, which shows the change in crystallite size distributions with reaction time at a low and a high temperature in a hypothetical system.

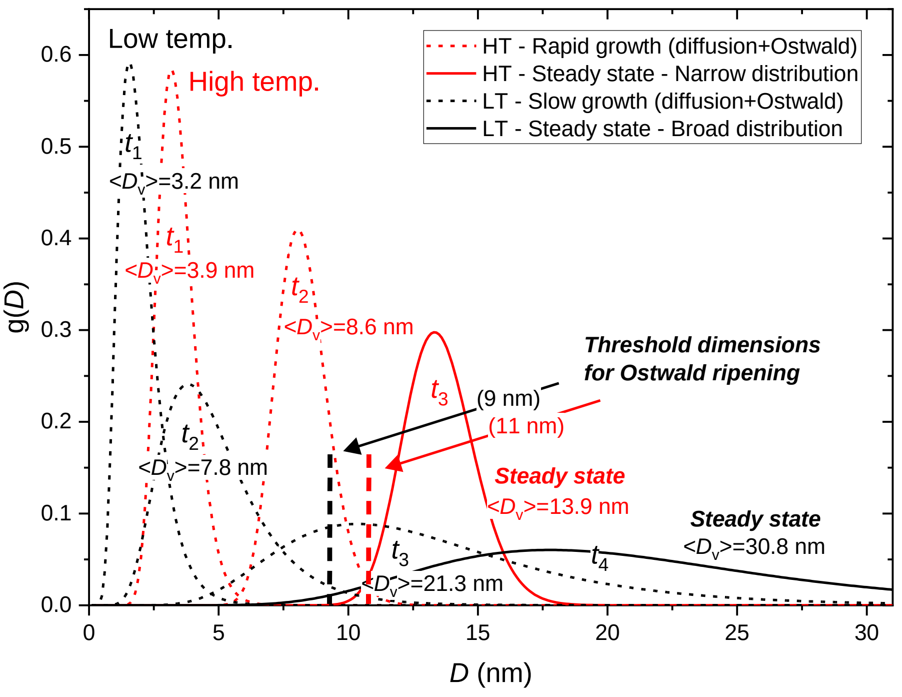

Figure 1: Evolution in lognormal crystallite size distributions and mean volume-weighted crystallite sizes, <D_v> at low (black) and high (red) temperature in a hypothetical system with critical threshold dimension for Ostwald ripening at 9 nm at low temperature and 11 nm at high temperature.

Here, a lognormal distribution of sizes is used, as this often provides a more physically meaningful description of the polydispersity in real nanoparticle systems. For the low temperature case, following the initial nucleation of primary nuclei, the supersaturation level drops and only permits further crystallization through incorporation of precursor material from solution onto existing grains (growth by diffusion). Simultaneously, the crystallites may grow through the dissolution of unstable smaller crystallites and the recrystallisation of the material onto larger stable ones (Ostwald ripening). The contribution of this growth mechanism is only significant when part of the crystallites in the system are below the characteristic (in)stability threshold size for the given temperature, which will be smaller at lower temperatures. As growth continues at low temperature by the combined mechanisms, a skewing of the size distribution takes place as it is energetically more favorable for material to grow onto larger grains. When the concentration of the precursor drops below the critical level for crystallization and all the crystallite sizes are above the threshold level for Ostwald ripening, the crystallization and growth stops, and steady state is attained. As a result of the inhomogeneous reaction, the obtained lognormal distribution of sizes may exhibit a volume-averaged mean size far above the mode, median and numeric mean of the distribution. For example, the steady state low temperature distribution plotted in Figure 1, has a mode of 17.8 nm, a number-averaged mean size of 21.4 nm but volume-averaged mean size of 30.8 nm. When heating rapidly to higher reaction temperatures, a big burst of homogeneous nucleation precipitates many primary nuclei occurs. This is followed by rapid growth mainly by diffusion until the precursor solution is no longer supersaturated. Due to the large number of homogeneously formed nuclei, a much narrower size distribution is obtained. If the sizes of all crystallites are above the Ostwald threshold, steady state may already be achieved, otherwise the growth will continue by Ostwald ripening until all crystallites are above the limit. Consequently, in this hypothetical example, the broad distribution with a $\langle D_v \rangle$ of 21.3 nm after t_3 at low temperature, will continue to grow by Ostwald ripening, while the distribution with a $\langle D_v \rangle$ of 13.9 nm after t_3 at high temperature will have reached steady state. The presence and contribution of equivalent mechanisms can thus explain the absence of Ostwald ripening in the seemingly smaller crystallites at higher reaction temperatures in the $NiFe_2O_4$ experiments.
